# Standardized Ileal Amino Acid Digestibilities in Different Corn Sources for Medium-Growing Yellow-Feathered Broilers and Their Prediction Models

**DOI:** 10.3390/ani16213330

**Published:** 2026-10-22

**Authors:** Wenpeng Chen, Qianwen Yuan, Xin Du, Jiali Long, Liyang Zhang, Shengchen Wang, Xiaoyan Cui, Xugang Luo

**Affiliations:** 1 Poultry Mineral Nutrition Laboratory, College of Animal Science and Technology, Yangzhou University, Yangzhou 225000, China; 2 Key Laboratory of Animal Nutrition and Feed Science of the Ministry of Agriculture, Wens Foodstuff Group Co., Ltd., Yunfu 527400, China; 3 State Key Laboratory of Animal Nutrition and Feeding, Mineral Nutrition Research Division, Institute of Animal Science, Chinese Academy of Agricultural Sciences, Beijing 100193, China

**Keywords:** corn, chemical composition, medium-growing yellow-feathered broiler, standardized ileal amino acid digestibility, prediction model

## Abstract

The present study first aimed to analyze the chemical compositions and amino acid (AA) profiles in 10 different sources of corn samples, then determine the SIAADs of these corn samples in medium-growing yellow-feathered broilers and establish rapid prediction equations for SIAADs based on their chemical compositions and AA profiles, and finally preliminarily assess their predictive performance. Broilers (*n* = 276; medium-growing yellow-feathered broilers; 63 days of age) were randomly allocated to 11 dietary treatments based on initial body weight. The experimental groups consisted of one nitrogen-free diet (NFD) control and 10 corn diet groups, and each experimental group consisted of 6 replicates, with 6 chickens per replicate in the control group and 4 chickens per replicate in each corn diet group. The experimental period was 5 days. At 67 days of age, ileal digesta samples were collected. Both apparent ileal amino acid digestibilities (AIAADs) and SIAADs in corn were evaluated. Predictive equations for corn SIAAD values were constructed via stepwise regression based on 9 corn samples. One independent corn sample was randomly selected to provide a preliminary assessment of predictive performance of the SIAAD models. The results showed that corn sources affected (*p* < 0.05) the AIAADs and SIAADs in yellow-feathered broilers. Among all assayed AAs, leucine (Leu) showed the highest apparent ileal digestibility (AID) (84.9%) and tryptophan (Trp) the lowest (52.8%), whereas glutamic acid (Glu) demonstrated the highest standardized ileal digestibility (SID) (94.6%) and Trp the lowest (64.5%). Equations predicting SID values for 6 AAs, including methionine (Met), phenylalanine (Phe), histidine (His), Trp, Glu, and proline (Pro), in corn samples for yellow-feathered broilers were constructed (*R*^2^ = 0.559 to 0.883, *p* ≤ 0.021). Among the established prediction models, the model for the SID of Trp showed the best fit (*R*^2^ = 0.883, *p* < 0.002), whereas the model for His SID showed the weakest goodness of fit (*R*^2^ = 0.559, *p* = 0.021). Except for the SID prediction model for Trp, which showed poor predictive accuracy, the other 5 SIAAD prediction models provided preliminary evidence supporting the potential applicability of these models. Therefore, these established prediction equations can provide an important reference for the rapid prediction of the SIDs of these AAs in corn samples for medium-growing yellow-feathered broilers in the future. Owing to the small sample size in this study, further independent external validation is necessary to verify the accuracy of the derived SIAAD prediction equations.

## Simple Summary

Standardized ileal amino acid digestibility (SIAAD) data of white-feathered broilers constitute the primary source of SIAAD reference data in the current literature, whereas corresponding data for yellow-feathered broilers remain scarce. Accordingly, we quantified SIAAD coefficients across multiple corn varieties in medium-growing yellow-feathered broilers and established predictive algorithms for rapid assessment of corn SIAAD. The main novelty lies in the establishment of corn SIAAD predictive models specifically for yellow-feathered broilers, which complements the deficient local SIAAD database. The above results can provide theoretical support for implementing precise feeding regimens in yellow-feathered broilers, thereby improving feed efficiency and supporting the sustainable development of poultry production. Nevertheless, this study is limited by a small sample size. Future research will include more corn cultivars and increase the sample size to improve result reliability.

## 1. Introduction

Corn serves as a major energy source in poultry rations owing to its ample yield, favorable taste, superior nutrient digestibility and high metabolizable energy (ME) [1,2]. It is a key component of broiler feed formulations in many countries and regions worldwide [3]. With the continued growth of the poultry industry, the demand for energy feed ingredients, particularly corn, has increased substantially, resulting in feed resource shortages and a sustained rise in corn demand. While corn is widely recognized as a relatively uniform feed ingredient, its chemical composition can vary substantially [4,5]. Hence, rational utilization of corn resources can help to alleviate the shortage of energy feed ingredients.

To achieve this goal, accurate evaluation of standardized ileal amino acid digestibility (SIAAD) in commonly used feedstuffs is essential for formulating balanced diets for medium-growing yellow-feathered broilers [6]. Such evaluation not only supports the scientific formulation of diets that meet amino acid (AA) requirements but also reduces nitrogen emissions and mitigates feed resource shortages [3,7]. The SIAAD method is a well-established and widely applied approach for determining AA digestibilities in poultry [8,9,10]. Several studies have successfully applied this method to assess the SIAADs of commonly used feed ingredients (one corn sample, soybean meals, and one fresh fermented soybean meal) in yellow-feathered broilers [11,12,13]. Moreover, researchers have developed SIAAD prediction models of sorghums for growing-finishing pigs [14], wheat and corn for white-feathered broilers [15,16,17], and sorghum and soybean meal for yellow-feathered broilers [13,18] based on their chemical properties. However, there is currently no report on the establishment of SIAAD prediction models for corn samples in medium-growing yellow-feathered broilers. These corn SIAAD prediction models for white-feathered broilers are not directly applicable to yellow-feathered broilers, mainly because SIAAD values in corn samples differ across broiler breeds [11,17]. Previous research indicated that corn SIAAD values were consistently higher in fast-growing white-feathered broilers [17] than in yellow-feathered broilers [11] [96.4 vs. 90.5% for arginine (Arg), 91.1 vs. 86.1% for histidine (His), 94.0 vs. 87.3% for isoleucine (Ile), 95.2 vs. 92.4% for leucine (Leu), 90.5 vs. 78.2% for lysine (Lys), 95.6 vs. 93.2% for phenylalanine (Phe), 87.7 vs. 80.6% for threonine (Thr), 93.1 vs. 88.6% for valine (Val), 94.6 vs. 91.8% for alanine (Ala), 92.8 vs. 87.5% for aspartic acid (Asp), 95.6 vs. 92.6% for glutamic acid (Glu), 89.9 vs. 80.3% for glycine (Gly) and 92.2 vs. 88.6% for serine (Ser)]. Moreover, the growth rate, environment, and variety of white-feathered broilers are different from those of yellow feathered broilers [2]. As for these considerations, establishing corn SIAAD prediction models tailored to yellow-feathered broilers represents a critical research priority. Notably, no published study has yet addressed this topic in yellow-feathered broilers.

Building on these previous findings, we hypothesized that corn SIAAD values for yellow-feathered broilers could be reliably predicted using corn chemical composition and AA profiles. To test this hypothesis, we collected corn samples from 10 different origins. We firstly determined their chemical composition and AA profiles, then measured SIAAD values of these corn samples in medium-growing yellow-feathered broilers, constructed predictive models for rapid SIAAD estimation using corn chemical components as predictors, and finally preliminarily assessed the developed models using one held-out corn sample.

## 2. Materials and Methods

### 2.1. Animal Ethics

All procedures concerning animal use and care were conducted in conformity with the requirements set forth by the Animal Use Committee under China’s Ministry of Agriculture (Beijing, China). Prior to initiation of the experiment, ethical approval was obtained from the Ethics Review Committee of the Department of Animal Science and Technology at Yangzhou University (License: SYXK (Su) 2021-0027).

### 2.2. Experimental Design and Treatments

This trial was arranged in a completely randomized design, and 11 dietary treatments were set in total, including one nitrogen-free diet (NFD) as the control and 10 treatment groups using corn samples from different origins as the sole source of crude protein (CP) and AAs.

### 2.3. Corn Samples, Animals and Diets

In total, 10 corn samples from different geographic origins (designated A1, A2, …, A10) were collected for evaluation. Detailed information on the origin of each sample is presented in Table 1.

A total of 300 healthy, uniform male Tianluma broilers aged 60 days were obtained from Huai’an Wen’s Livestock Co., Ltd. (Huai’an, China). The chickens were weighed after an overnight fast to ensure that their weights met the 60-day-old weight requirement for medium-growing yellow-feathered broilers. Subsequently, 276 broilers exhibiting comparable body weight (mean = 1.33 kg/bird) were screened for the trial [13,18]. Randomization was performed at the cage level. Broilers that met the inclusion criteria were firstly stratified by body weight to ensure a balanced distribution across groups and then randomly allocated to cages. The selected chickens were randomly assigned to 11 treatment groups: one nitrogen-free diet (NFD) group and 10 experimental corn diet groups. The NFD group was assigned to 6 replicate cages with 6 birds per cage, while each corn diet treatment had 6 replicate cages containing 4 birds apiece. Due to the low feed intake observed in the NFD group, terminal ileal digesta collection might have been insufficient. Therefore, the number of birds per replicate in this group was increased from 4 to 6 to ensure an adequate sample size for experimental analyses. Environmental conditions during the experimental period, including ambient temperature, relative humidity, and lighting duration, were regulated in strict compliance with the industry standards established for optimal growth and management of yellow-feathered broilers. Birds were housed in stainless steel cages measuring 90 × 70 × 45 cm (length × width × height) equipped with feeders and drinkers. Throughout the pre-experimental adaptation period (60–62 days of age), all birds received ad libitum access to a standardized commercial pelleted basal diet and drinking water.

Since 1-day-old broilers are in an early growth stage and their digestive organs are not yet fully mature, 63- to 67-day-old broilers were selected for the formal experimental period. From 63 days of age, birds had ad libitum access to their respective treatment diets in a pelleted form and tap water for an additional 5 days. Daily feed intake on a replicate basis did not differ (*p* > 0.05) across different dietary treatments. In the tested corn diets, the evaluated corn samples served as the sole source of CP and AAs, and were combined with vitamin-mineral premixes. Details of the chemical composition analysis of all 10 corn samples are provided in Table 2. The NFD was composed of corn starch, dextrose, and vitamin-mineral premixes. As an inert marker, titanium dioxide (TiO_2_) was incorporated at an inclusion level of 0.4% into both the NFD and all test diets. Table 3 lists the composition and nutrient contents of all 11 diets. The concentrations of all other nutrients except for AAs and CP in all diets met the nutrient requirements for the corresponding growth stage of yellow-feathered broilers in China [19]. Feed intake was recorded daily on a replicate basis. For each replicate, total feed intake during the experimental period was calculated as the amount of feed offered minus the residual feed remaining after feeding.

### 2.4. Sample Collections and Preparations

Representative samples of corn samples and experimental diets were mechanically comminuted with a stainless-steel mill (Yanshanzhengde Inc., Beijing, China) and passed through a 0.45-mm sieve to achieve uniform particle size distribution prior to chemical composition analysis.

At 67 days of age, all birds were humanely euthanized via carbon dioxide inhalation while in a postprandial state. In this study, animals were not subjected to a pre-collection fasting period and were maintained under normal feeding conditions throughout the experimental period. Following isolation of the ileum (extending from the vitelline diverticulum to 2 cm proximal to the ileocecal junction), digesta were recovered from the distal ileal segment via distilled water lavage. The collected ileal digesta were immediately stored at −20 °C, and then lyophilized using a freeze dryer. Freeze-dried ileal digesta samples from all birds within each replicate were thoroughly homogenized, finely ground, and stored at −20 °C for subsequent analyses.

### 2.5. Measurement Indicators and Methods

Proximate composition parameters, including CP, moisture, ether extract (EE), crude fiber (CF) and crude ash (CA) in corn and diet samples were analyzed according to AOAC International methods (976.05, 934.01, 920.39, 962.09, 942.05, respectively) [20]. The contents of neutral detergent fiber (NDF) and acid detergent fiber (ADF) in corn samples were measured according to AOAC International official methods 973.18 and 993.19, respectively [8,20,21,22]. Phytic acid (PA) content in corn samples was determined using a kit (catalog number SAP-2-Y; Suzhou Keming Biotechnology Co., Ltd., Suzhou, China). Starch (ST) content in corn was determined by the kit (catalog number BC0700; Beijing Solarbio Science & Technology Co., Ltd., Beijing, China). The AA composition of corn, diet, and digesta samples was determined by high-performance liquid chromatography (HPLC) following acid hydrolysis, according to AOAC International method 982.30 [20]. The TiO_2_ content in diet and digesta samples was determined using the spectrophotometric procedures of Titgemeyer et al. [23].

### 2.6. Calculations

The ileal endogenous amino acid (IEAA) losses of broilers were calculated using the data from the NFD group, referring to the method reported by Cao et al. [8] and Yun et al. [16]. The calculation formula is as follows:

IEAA (mg/kg of DMI) = (W_I_ × W_II_)/W_III_

In this formula, W_I_ represents the AA content in ileal digesta of broilers fed the NFD (mg/kg); W_II_ is the TiO_2_ content in the NFD (mg/kg); W_III_ stands for the TiO_2_ content in ileal digesta of birds in the NFD group (mg/kg).

Apparent ileal amino acid digestibilities (AIAADs) and SIAADs were calculated with reference to Wang et al. [24], and the formulas are shown below:

AIAAD (%) = 100 − (D_I_ × D_II_)/(D_III_ × D_IV_) × 100

SIAAD (%) = AIAAD + IEAA/D_III_ × 100

where D_I_ is the AA concentration in ileal digesta of broilers fed corn-based diets; D_II_ is the TiO_2_ concentration in the experimental diet; D_III_ represents the AA concentration in the corresponding experimental diet; D_IV_ is the TiO_2_ concentration in ileal digesta of the corn diet groups. All concentrations are expressed on a mg/kg dry matter basis.

### 2.7. Statistical Analyses

Data for feed intake, AIAADs and SIAADs were analyzed using one-way analysis of variance (ANOVA) and the general linear model (GLM) procedure in SAS version 9.4 (2013) (SAS Institute, Cary, North Carolina), and each replicate cage was taken as the independent experimental unit. The threshold of statistical significance was set at *p* < 0.05 for all tests. Additionally, Pearson product-moment correlation coefficients were computed to quantify the strength of linear associations between chemical composition, AA profiles, and SIAAD values determined from the 10 corn samples. Significant differences among treatments were determined using the least significant difference (LSD) method [25]. Based on recent relevant studies [13,18], using data from 9 corn samples (A1, A2, A4~A10), stepwise regression analysis was used to develop predictive equations for SIAADs, and optimal models were selected according to adjusted *R*^2^ (coefficient of determination), RMSE (root mean square error), and biological plausibility of predictors. To assess the accuracy of SIAAD prediction equations, corn sample A3 was randomly selected via the RANDGEN subroutine in SAS, and its measured chemical compositions and AA profiles were used as inputs for a preliminary assessment of the models.

## 3. Results

### 3.1. Chemical Constituents of 10 Corn Samples

The chemical compositions of the corn samples are presented in Table 2 (air-dry basis). Among the 10 different corn sources, variations were observed in their nutritional profiles, with moisture fluctuating from 11.61 to 14.13%, CP from 6.75 to 9.18%, EE spanning 2.50 to 3.58%, CA varying from 0.99 to 1.55%, CF ranging from 1.22 to 2.29%, NDF between 7.15 to 12.32%, ADF from 1.10 to 2.94%, PA content ranging from 0.85 to 1.11%, starch levels 59.04 to 69.58%, and NFE ranging from 70.98 to 75.64%. The coefficients of variation (CVs) for moisture, ST, and NFE were small, 7.05%, 6.42%, and 2.00%, respectively, and the CVs for CF, NDF, and ADF content were large, 19.62%, 17.62%, and 23.24%, respectively. The ten corn sources exhibited diverse essential amino acid (EAA) profiles, with concentration ranges presented as follows: Val ranged from 0.35% to 0.48%, while methionine (Met) demonstrated a notably wider range, varying from 0.07% to 0.24%. Ile ranged from 0.22% to 0.31%, Leu from 0.85% to 1.24%, Thr from 0.26% to 0.37%, Phe from 0.34% to 0.49%, Lys from 0.21% to 0.29%, His from 0.19% to 0.26%, Arg from 0.36% to 0.51%, and tryptophan (Trp) from 0.04% to 0.05%. The ten corn sources exhibited diverse non-essential amino acid (NEAA) profiles, with concentration ranges presented as follows: Asp ranged from 0.46 to 0.63%, Ser from 0.35 to 0.49%, Glu from 1.29 to 1.96%, Gly from 0.26 to 0.38%, Ala from 0.54 to 0.77%, cysteine (Cys) from 0.07 to 0.19%, tyrosine (Tyr) from 0.21 to 0.31%, and proline (Pro) from 0.77 to 1.10%. Large variations in CV were observed for individual AAs, with EAAs demonstrating CVs ranging from 9.44% for Val (lowest) to 46.29% for Met (highest). NEAAs exhibited comparable variation patterns, with coefficients of variation spanning from 11.20% for Ala (lowest) to 40.85% for Cys (highest).

### 3.2. Determination of AIAAD in Corn from 10 Different Sources Using Medium-Growing Yellow-Feathered Broilers

Table 4 shows the AIAADs of different corn sources for medium-growing yellow-feathered broilers. The corn sources had an effect (*p* < 0.05) on the AIAADs of all 18 AAs. For EAAs, the AID showed marked variation across individual AAs. The AID ranged from 51.0 to 68.4% for Val, 80.0 to 88.8% for Met, 67.4 to 78.5% for Ile, 78.6 to 88.6% for Leu, 52.1 to 65.1% for Thr, 65.9 to 78.5% for Phe, 55.9 to 69.5% for Lys, 73.6 to 84.6% for His, 67.8 to 78.2% for Arg, and 42.8 to 60.2% for Trp. Among them, the mean AID of Leu was the highest at 84.9% and the AID of Trp was the lowest at 52.8%. For NEAAs, obvious disparities in AID were observed among different AAs. The AID ranged from 59.3 to 72.9% for Asp, 60.6 to 74.8% for Ser, 78.0 to 86.6% for Glu, 61.8 to 72.9% for Gly, 77.9 to 85.9% for Ala, 63.5 to 76.3% for Cys, 77.8 to 87.7% for Tyr, and 64.8 to 83.7% for Pro. Among them, the mean AID of Glu was the highest at 83.5% and the AID of Asp was the lowest at 66.6%. The CVs of EAA AIDs were 8.15% for Val, 3.69% for Met, 5.87% for Ile, 3.52% for Leu, 7.21% for Thr, 5.66% for Phe, 6.75% for Lys, 4.55% for His, 4.49% for Arg, and 9.42% for Trp. Leu exhibited the most consistent AID, whereas Trp demonstrated comparatively lower consistency in AID. The CVs of NEAA AIDs were 6.63% for Asp, 7.34% for Ser, 3.60% for Glu, 4.91% for Gly, 3.47% for Ala, 5.32% for Cys, 3.74% for Tyr, and 7.01% for Pro. Ala exhibited the most consistent AID, whereas Ser demonstrated comparatively lower consistency in AID.

### 3.3. Determination of SIAAD in Corn from 10 Different Sources Using Medium-Growing Yellow-Feathered Broilers

Table 5 summarises all SIAAD data. Corn sources had no effect (*p* > 0.05) on the SIDs values of 9 AAs, including Ile, Leu, Thr, Lys, Ser, Gly, Ala, Cys, and Tyr. However, the SID of the remaining nine AAs, including Val, Met, Phe, His, Arg, Trp, Asp, Glu, and Pro were affected (*p* < 0.05). The mean SID values of the 18 AAs ranged from 64.5% to 94.6%. For EAAs, the SID presented noticeable differences between separate AAs. The SID ranged from 86.4 to 96.9% for Val, 90.1 to 97.2% for Met, 86.1 to 96.1% for Ile, 88.8 to 97.2% for Leu, 86.2 to 92.8% for Thr, 87.3 to 95.4% for Phe, 82.9 to 93.7% for Lys, 83.5 to 92.6% for His, 89.8 to 95.6% for Arg, and 54.8 to 72.2% for Trp. Among them, the mean SID value of Leu was the highest at 94.0% and that of Trp was the lowest at 64.5%. For NEAAs, the SID exhibited marked variation across individual AAs. The SID ranged from 88.3 to 97.7% for Asp, 88.4 to 96.2% for Ser, 90.3 to 97.5% for Glu, 81.8 to 88.9% for Gly, 90.1 to 97.1% for Ala, 82.7 to 91.4% for Cys, 89.1 to 96.6% for Tyr, and 75.6 to 92.1% for Pro. Among them, the mean SID value of Glu was the highest at 94.6%, and that of Gly was the lowest at 85.5%. Variation in SIAAD differed markedly between nutritional categories. For EAAs, the CV ranged from 2.30% (Arg) to 7.75% (Trp), whereas NEAAs demonstrated CVs between 2.33% (Ala) and 5.51% (Pro). Considering all 18 AAs, CV values for digestibility spanned from 2.19 to 7.75%, with Arg exhibiting minimal fluctuation (2.19%) and Trp showing maximum fluctuation (7.75%).

### 3.4. Correlations Among Chemical Compositions, AA Profiles, and SIAADs of Corn Samples for Medium-Growing Yellow-Feathered Broilers

Pearson correlation analysis was conducted to investigate the relationships among chemical composition, AA profiles, and SIAAD among the ten corn samples evaluated in medium-growing yellow-feathered broilers, with results presented in Table 6. The SID values of Ser (r = 0.743, *p* < 0.05) and Cys (r = 0.772, *p* < 0.05) were positively correlated with CA. Except for the SID values of Val, Ile, Thr, Lys, Arg, Trp, Asp, and Ser, the SID values of the remaining 10 AAs in corn samples showed negative correlations (r = −0.919 to −0.735, *p* < 0.05) with PA. Table 7 and Table 8 show the correlations among EAA and NEAA contents and SIAAD values in corn for medium-growing yellow-feathered broilers. The SID of Trp had a negative correlation with Met (r = −0.773, *p* < 0.05) and Cys (r = −0.809, *p* < 0.05), and the SID of Thr had a positive correlation with Lys (r = 0.864, *p* < 0.05). The remaining positive and negative correlations were not statistically significant.

### 3.5. Predicting SIAAD Values in Corn Samples via Chemical Compositions and AA Characteristics for Medium-Growing Yellow-Feathered Broilers

The results of the prediction models are shown in Table 9. Six prediction models for SIAADs in corns were successfully developed for medium-growing yellow-feathered broilers (*R*^2^ = 0.559 to 0.883, *p* ≤ 0.021). The main predictors in the equations included CF, PA, ADF, CP and Cys. Furthermore, the prediction model for the SID of Trp exhibited the best fit (*R*^2^ = 0.883, *p* < 0.002), while that for the SID of His demonstrated the lowest fitted goodness (*R*^2^ = 0.559, *p* = 0.021). The SID prediction models for Trp and Pro attained *R*^2^ > 0.80. These models showed high in-sample *R*^2^ values; however, these values did not establish predictive reliability or robustness.

### 3.6. Preliminary Assessment of SIAADs Prediction Models in Corn Samples for Medium-Growing Yellow-Feathered Broilers

SID prediction models developed in the current study were applied to corn sample A3 using the measured chemical composition and AA profile data as inputs, generating predicted SID values for the 6 AAs analyzed (Met, Phe, His, Trp, Glu and Pro). The predicted SID values for corn A3 are summarized in Table 10, allowing assessment of model applicability and prediction accuracy. For corn sample A3, the deviations between measured and model-predicted SID values of these AAs were 0.4% for Met, 0.9% for Phe, 1.2% for His, 4.6% for Trp, 0.4% for Glu, and 2.1% for Pro. Apart from Trp, the predicted SID values for the remaining AAs fell within the range of the measured values ± SD, confirming good prediction accuracy.

## 4. Discussion

The results of this study indicate that chemical composition (CF, PA, ADF and CP) and AA profile (Cys) can be used to establish prediction equations for SIDs of 6 AAs (Met, Phe, His, Trp, Glu and Pro) in corn samples for yellow-feathered broilers. The predicted SID value of Trp deviated from the acceptable range of its experimental measurement ± SD; in addition, prediction models for the SIDs of Val, Arg, and Asp were not established, although their SID values were significantly affected by corn source. For Ile, Leu, Thr, Lys, Ser, Gly, Ala, Cys, and Tyr, prediction models were not established because their SID values were not significantly affected by corn source. These findings support our hypothesis, while providing a solid theoretical basis for the rapid prediction of SIAAD values in corn samples for yellow-feathered broilers, as well as for precisely supplementing AAs in corn-based diets for yellow-feathered broilers.

In the current study, based on the analysis of conventional chemical compositions in 10 corn samples from different sources, it was found that the coefficients of variation for most chemical indicators exceeded 10%, except for moisture, PA, ST, and NFE. The EE content in corn samples was relatively lower compared with the findings reported by An et al. [26]. The average contents of CF and ADF were 1.72% and 2.27%, respectively, which were similar to the values determined by Pan et al. [27] for 10 corn samples. The mean contents CA and NDF were 1.19% and 8.77%, respectively, which were close to the 1.4% and 9.22% reported by Li et al. [28]. The mean CP content of corn from different origins was 8.02%, slightly lower than the 8.30% reported by Liu et al. [29]. Moreover, the mean ST content was 64.95%, lower than the 72.77% reported by Li et al. [30]. Such differences may be largely attributed to variations in corn origins, growing conditions, cultivars, and storage environments [31,32]. Among the 18 AAs analyzed in this study, Met and Cys exhibited high CVs of 46.29% and 40.85%. The relatively high CVs observed for Met and Cys may reflect both analytical variability associated with acid hydrolysis and HPLC determination and inherent differences in the AA composition of the maize samples. This high variability was consistent with the findings reported by Xiao et al. [33], indicating pronounced variation in these two AAs. Most of the AA contents in corn were the same or close to those reported in the literature [26,34]. The different corn varieties selected in the present study exhibited considerable variation in chemical compositions and AA contents, indicating that the corn from different sources used in this study were highly representative, and thereby enabling the development of reliable regression models.

The mean AIAAD values for corn from ten different sources ranged from 52.8% for Trp to 84.9% for Leu. An et al. [26] reported that Leu exhibited the highest AID among all AAs in white-feathered broilers, which is consistent with our findings. The AID values of His, Ser, Glu, and Pro were closely aligned with those observed by Cowieson et al. [7] in 28-d-old white-feathered broilers, while the AID values of a few AAs differed considerably. AIAAD does not account for basal endogenous losses (BELs) of AAs, resulting in inaccurate estimates. Therefore, accurate assessment of the SIAADs in corn is essential for formulating balanced diets for yellow-feathered broilers. Results from the present study demonstrated that corn samples from different sources exhibited significant differences in the SID values of His, Arg, Trp, Val, Met, Phe, Asp, Glu, and Pro for yellow-feathered broilers. The mean SIAAD values of corn from 10 different sources in yellow-feathered broilers ranged from 64.5% for Trp to 94.6% for Glu. The values were comparable to the SIAAD ranges in corn for 13-d-old or 21-d-old white-feathered broilers as reported by Barua et al. [2] and Hang et al. [17]. The lowest SID of Trp in this study is consistent with the findings of Yuan et al. [13] in medium-growing yellow-feathered broilers fed soybean meal. The highest Trp SID variability observed here is also consistent with the report of Zuber et al. [35] for corn Trp SID variability in de-appendaged laying hens. The Trp content in corn is extremely low, thus the proportion of endogenous AA losses and detection errors in the measured value is significantly higher than that of other AAs, leading to lower digestibility and greater variation. Nevertheless, the SID values of Ile, Phe, His, Arg, Trp, Gly, and Pro reported by Hang et al. [17] were markedly higher than the observations of the present study. In addition, Liu et al. [11] determined SIAAD in a single corn sample for yellow-feathered broilers, and the results indicated that its SIAAD values ranged from 78.2% for Lys to 93.9% for Met. Val, Thr, Lys, and Gly showed lower SID values relative to this study, the remaining AAs (Phe, His, Arg, Asp, Met, Ile, Leu, Ser, Glu, Ala, Cys, and Pro) exhibited similar SID values. These differences may be attributed to variations in broiler breed and age, corn breed and source, and the number of corn samples [2]. Additionally, CP contents across rations A1-A10 ranged from 6.61% to 8.87%, which may have affected the feed intake of birds since birds were fed ad libitum. However, daily feed intake on a replicate basis did not differ significantly across different dietary treatments, suggesting that feed intake would be unlikely to have substantially affected ileal AA digestibility values.

Correlation analyses in the present study indicate that the SIDs of Leu, Phe, His, Glu, Gly, Ala, Cys, Tyr, and Pro in corn samples were negatively correlated with PA, which is consistent with the findings in 13-d-old white-feathered broilers reported by Hang et al. [17]. As a significant antinutritional factor, PA increases mucin secretion and IEAA losses in the chicken gastrointestinal system, as reported by Onyango et al. [36], and impairs AA digestibility in broilers [37]. PA was retained as an important explanatory variable in most models due to its variation among corn samples and its reported inhibitory effect on AA digestibility in corn samples. A significant positive association was observed between CA and the SIDs of Ser and Cys in the present study, aligning with the observations of Pan et al. [27], who found positive correlations of the SIDs of Thr, Ser, Ala, and Cys for laying hens with CA in corn. Additionally, the SID of Thr was positively correlated with Lys, which is contrary to the findings of Hang et al. [15] in 28-d-old white-feathered broilers, possibly due to different broiler breeds and ages between the above studies. A negative correlation of the SID of Trp with Met or Cys as observed in the present study has not been reported in other studies. The above results suggest that PA, CA, and specific AA pairs may affect the SIDs of some AAs in corn for poultry.

To improve the accuracy of diet formulation and better meet the AA needs of yellow-feathered broilers, it is necessary to develop prediction models for the SIAADs of corn ingredients [38]. The development of prediction models for SIAADs based on the chemical compositions and AA profiles of corn is an important step toward rapidly estimating corn SIAADs for yellow-feathered broilers. In the present study, 6 SIAAD prediction models incorporating chemical compositions (CF, PA, ADF and CP) and AA profile (Cys) were successfully developed. The signs of the regression coefficients indicate the direction of their association with SID. For example, the negative coefficient for PA is biologically plausible, as phytate may interact with proteins and reduce AA digestibility. Notably, the *R*^2^ values for the SID prediction models of Trp and Pro all exceed 0.80, indicating favourable fitting performance on the present dataset. Despite of its good fitting statistics (*R*^2^ = 0.883 and RMSE = 0.02), the Trp prediction model showed relatively poor agreement between the predicted and measured values for corn sample A3. This discrepancy raises a concern about potential overfitting and indicates that high fitting performance may not necessarily reflect the model’s predictive capability for independent samples. Therefore, based on the current evidence, the prediction model for Trp SID should not be considered suitable for practical application at this stage. In line with the *R*^2^ results, the RMSE values varied substantially among different AA indices. Several high-performance models achieved excellent predictive precision: the equations for SID Trp (RMSE = 0.02) yielded extremely low RMSE, corresponding well to their high coefficient of determination. By contrast, SID His (RMSE = 2.25) and SID Glu (RMSE = 1.52) exhibited relatively higher RMSE, indicating that the selected predictor variables had limited predictive capacity for these two AAs. Currently, the prediction equations for SIAADs in pigs or white-feathered broilers have been established based on the chemical compositions of feed ingredients [14,17,39,40]. However, corresponding SIAAD prediction equations for corn have not been reported in yellow-feathered broilers to date. Hang et al. [17] developed and validated SIAAD prediction models for 13-d-old white-feathered broilers, incorporating corn chemical compositions and AA profiles as predictor variables. To be precise, crude-protein content served as a favourable predictive factor, while PA and ADF exerted adverse predictive effects. This finding accords well with our current experimental results. In addition, phytase supplementation hydrolyzes phytic acid, releasing bound AA and minerals and improving AA and mineral digestibilities. Therefore, adding phytase is expected to lessen the negative impact of PA on AA digestibility and thus might influence these prediction models in practical field applications.

The accuracies of the SIAAD prediction models need to be validated in order to be applied to practical production. A previous study showed the predictive accuracy of the equation by comparing the differences (bias) and the standard deviation of the differences between measured and predicted values [41]. To assess the predictive accuracy of the developed regression models, one corn sample, A3, was randomly selected as an independent sample for preliminary assessment of SIAAD prediction performance. Except for the prediction model for the SID of Trp in corn samples, which showed poor predictive accuracy, the remaining 5 models for the SIDs of Met, Phe, His, Glu, and Pro provided preliminary evidence supporting their potential applicability. According to Yuan et al. [13] and Cui et al. [18], who respectively studied soybean meals and sorghums, the accuracy of the established prediction model was validated using data from a single raw-material source, with favorable results. Because a single-sample preliminary assessment cannot capture batch variability or generalizability due to genetics, environment, processing or measurement, such an assessment might overstate generalizability. Therefore, external validation with more corn samples across batches and conditions is needed to assess accuracy and applicability. We plan to test multiple corn lots in the future to improve transferability and robustness. On the other hand, the relatively small number of corn samples (*n* = 9) may have led to the risk of overfitting, especially in models with more independent variables and extremely high *R*^2^. We acknowledge that more external validations in independent broiler cohorts are needed to confirm generalizability and to further assess the risk of overfitting in these models.

The main strengths of this study are the construction of SIAAD prediction equations of corn samples for medium-growing yellow-feathered broilers, which supplements the AA digestibility database for this specific breed and provides a quantitative reference for precise feed formulation. Limitations include the limited corn sample size, which restricts the generalizability and robustness of the models, and the insufficient assessment strategy: the models have only undergone preliminary assessment with the existing dataset, with no independent in vivo validation trials conducted. Thus, their predictive reliability across different production environments and raw material batches requires further confirmation. Future research will expand the diversity and sample size of corn materials, and optimize the validation system through in vivo tests, to improve the models’ practical application value in production.

## 5. Conclusions

This investigation successfully developed SID regression prediction equations for 6 AAs in corn grains for medium-growing yellow-feathered broilers. These established equations can provide an important reference for the rapid predictions of the SIAADs in corn samples for yellow-feathered broilers based on their chemical compositions (CF, PA, ADF and CP) and AA profile (Cys). These findings provide a scientific basis for precision feeding programs for medium-growing yellow-feathered broilers, helping to elevate feed efficiency and promote sustainable poultry production. Nevertheless, several limitations exist in the present study. For example, the accuracy of the prediction model was preliminarily assessed using only one held-out corn sample. More importantly, the relatively limited sample size may have introduced potential bias into the models. Accordingly, subsequent investigations will expand the sample size and incorporate multiple corn cultivars and geographical growing origins to minimise experimental errors and strengthen the reliability of experimental datasets.

## Figures and Tables

**Table 1:** Sources of the 10 tested corn samples.

Number	Sources	Number	Sources
A1	Ningxia	A6	Neimenggu
A2	Shanxi	A7	America
A3	Jilin	A8	Liaoning
A4	Henan	A9	Xinjiang
A5	Jilin	A10	Shandong

**Table 2:** Analyzed chemical compositions in 10 tested corn sources (air-dry basis) ^1^.

Item (%)	Corn Sources										Mean	SD	CV (%)
	A1	A2	A3	A4	A5	A6	A7	A8	A9	A10			
Moisture	11.67	12.40	11.61	13.89	13.89	12.88	12.30	14.13	12.65	13.21	12.86	0.91	7.05
CP	6.75	8.52	8.20	7.36	7.05	7.42	7.71	9.18	8.91	9.07	8.02	0.88	10.98
EE	2.89	2.96	3.16	3.30	3.56	2.50	3.54	3.01	2.89	3.58	3.14	0.36	11.32
CA	1.03	1.08	1.21	1.23	0.99	1.26	1.17	1.25	1.11	1.55	1.19	0.16	13.28
CF	2.02	1.83	2.00	1.62	2.29	1.24	1.60	1.22	1.76	1.61	1.72	0.34	19.62
NDF	7.43	8.07	8.21	8.24	10.02	7.15	9.64	7.74	8.92	12.32	8.77	1.55	17.62
ADF	1.96	2.05	2.21	2.06	2.62	1.10	2.79	2.49	2.52	2.94	2.27	0.53	23.24
PA	0.94	0.91	0.95	0.93	0.90	0.90	1.11	0.91	0.96	0.85	0.94	0.07	7.36
ST	59.20	68.02	59.04	67.85	60.63	63.25	68.48	64.60	69.58	68.82	64.95	4.17	6.42
NFE	75.64	73.21	73.82	72.60	72.22	74.70	73.68	71.21	72.68	70.98	73.07	1.46	2.00
EAAs													
Val	0.43	0.40	0.37	0.48	0.37	0.39	0.39	0.35	0.39	0.43	0.40	0.04	9.44
Met	0.08	0.07	0.07	0.10	0.08	0.15	0.18	0.19	0.16	0.24	0.13	0.06	46.29
Ile	0.27	0.25	0.23	0.31	0.24	0.24	0.24	0.22	0.24	0.24	0.25	0.02	9.75
Leu	1.00	0.97	0.87	1.24	0.93	0.91	0.91	0.85	0.90	1.21	0.98	0.14	13.89
Thr	0.31	0.29	0.28	0.36	0.27	0.28	0.29	0.26	0.27	0.37	0.30	0.04	12.74
Phe	0.41	0.40	0.36	0.49	0.38	0.37	0.38	0.34	0.38	0.42	0.39	0.04	10.60
Lys	0.27	0.25	0.23	0.24	0.21	0.22	0.23	0.21	0.24	0.29	0.24	0.03	10.70
His	0.24	0.21	0.20	0.26	0.20	0.22	0.22	0.19	0.22	0.25	0.22	0.02	9.77
Arg	0.51	0.38	0.39	0.50	0.37	0.41	0.40	0.36	0.38	0.46	0.42	0.05	13.13
Trp	0.04	0.04	0.04	0.05	0.04	0.04	0.04	0.04	0.04	0.05	0.04	0.004	10.25
NEAAs													
Asp	0.60	0.55	0.49	0.63	0.47	0.51	0.52	0.46	0.51	0.63	0.54	0.06	11.55
Ser	0.42	0.40	0.37	0.49	0.36	0.37	0.39	0.35	0.35	0.44	0.40	0.05	11.48
Glu	1.61	1.55	1.40	1.96	1.36	1.43	1.46	1.29	1.37	1.67	1.51	0.20	13.07
Gly	0.33	0.30	0.29	0.38	0.27	0.29	0.29	0.26	0.29	0.38	0.31	0.04	13.60
Ala	0.64	0.63	0.57	0.77	0.60	0.58	0.59	0.54	0.59	0.71	0.62	0.07	11.20
Cys	0.07	0.07	0.07	0.09	0.07	0.14	0.16	0.16	0.16	0.19	0.12	0.05	40.85
Tyr	0.28	0.23	0.21	0.31	0.26	0.27	0.23	0.25	0.24	0.25	0.25	0.03	11.63
Pro	0.91	0.87	0.80	1.10	0.86	0.82	0.86	0.77	0.88	1.08	0.89	0.11	12.38

^1^ The value is the average of the two measured values. Abbreviations: SD, standard deviation; CV, coefficient of variation; CP, crude protein; EE, ether extract; CA, crude ash; CF, crude fiber; NDF, neutral detergent fiber; ADF, acid detergent fiber; PA, phytic acid; ST, starch; NFE, nitrogen-free extract; EAAs, essential amino acids; Val, valine; Met, methionine; Ile, isoleucine; Leu, leucine; Thr, threonine; Phe, phenylalanine; Lys, lysine; His, histidine; Arg, arginine; Trp, tryptophan; NEAAs, non-essential amino acids; Asp, aspartic acid; Ser, serine; Glu, glutamic acid; Gly, glycine; Ala, alanine; Cys, cysteine; Tyr, tyrosine; Pro, proline.

**Table 3:** Ingredient composition and nutrient levels of the NFD and 10 corn diets (as-fed basis).

Item	NFD	Tested Corn Diets									
		A1	A2	A3	A4	A5	A6	A7	A8	A9	A10
Ingredient, %											
Corn	-	95.66	95.66	95.66	95.66	95.66	95.66	95.66	95.66	95.66	95.66
Corn starch	63.81	-	-	-	-	-	-	-	-	-	-
Glucose ^1^	27.00	-	-	-	-	-	-	-	-	-	-
CaHPO_4_^1^	1.91	1.80	1.80	1.80	1.80	1.80	1.80	1.80	1.80	1.80	1.80
Limestone ^1^	1.16	1.30	1.30	1.30	1.30	1.30	1.30	1.30	1.30	1.30	1.30
NaCl ^1^	0.18	0.30	0.30	0.30	0.30	0.30	0.30	0.30	0.30	0.30	0.30
Cellulose ^1^	5.00	-	-	-	-	-	-	-	-	-	-
Choline chloride ^1^ (50%)	0.15	0.15	0.15	0.15	0.15	0.15	0.15	0.15	0.15	0.15	0.15
Sodium bicarbonate ^1^	0.15	0.15	0.15	0.15	0.15	0.15	0.15	0.15	0.15	0.15	0.15
Trace element premix ^1,2^	0.23	0.23	0.23	0.23	0.23	0.23	0.23	0.23	0.23	0.23	0.23
Antioxidant	0.01	0.01	0.01	0.01	0.01	0.01	0.01	0.01	0.01	0.01	0.01
TiO_2_	0.40	0.40	0.40	0.40	0.40	0.40	0.40	0.40	0.40	0.40	0.40
Total	100.00	100.00	100.00	100.00	100.00	100.00	100.00	100.00	100.00	100.00	100.00
Nutrient levels											
ME/(kcal·kg^−1^) ^3^	2848	3080	3080	3080	3099	3080	3080	3080	3080	3080	3099
CP ^4^, %	0.34	7.69	7.89	6.61	8.73	6.86	7.04	6.98	7.21	8.02	8.87
Lys ^4^, %	-	0.24	0.24	0.21	0.26	0.22	0.22	0.22	0.23	0.20	0.25
Met ^4^, %	-	0.15	0.16	0.14	0.21	0.15	0.14	0.15	0.16	0.12	0.20
Thr ^4^, %	-	0.26	0.27	0.23	0.31	0.24	0.25	0.24	0.25	0.24	0.30
Trp ^4^, %	-	0.05	0.05	0.05	0.07	0.05	0.05	0.05	0.05	0.04	0.07
Met + Cys ^4^, %	-	0.31	0.32	0.28	0.39	0.29	0.28	0.29	0.31	0.26	0.38
Ca ^3^, %	0.89	0.92	0.92	0.92	0.92	0.92	0.92	0.92	0.92	0.92	0.92
TP ^3^, %	0.38	0.65	0.65	0.65	0.66	0.65	0.65	0.65	0.65	0.65	0.66
Nonphytate P ^3^, %	0.37	0.44	0.44	0.44	0.45	0.44	0.44	0.44	0.44	0.44	0.45

^1^ Feed grade. ^2^ Provided per kilogram of diet: vitamin A 10000 IU; vitamin D_3_ 3750 IU; vitamin E 27.5 IU, vitamin K 2.5 mg; vitamin B_1_ 2.5 mg; vitamin B_2_ 8.0 mg; vitamin B_6_ 3.75 mg; vitamin B_12_ 0.025 mg; pantothenic acid calcium 12.5 mg; niacin 45 mg; folic acid 125 mg; biotin 0.125 mg; zeolite 1347.8 mg; Cu (CuSO_4_·5H_2_O) 7 mg; Fe (FeSO_4_·H_2_O) 80 mg; Zn (ZnSO_4_·H_2_O) 55 mg; Mn (MnSO_4_·H_2_O) 60 mg; Se (Na_2_SeO_3_) 0.15 mg; I (Ca(IO_3_)_2_·H_2_O) 0.5 mg. ^3^ Calculated values. ME values were calculated by weighted summation based on ingredient ME values and inclusion rates. ^4^ Measured values based on duplicate determinations. Abbreviations: NFD, Nitrogen-free diet; TiO_2_, titanium dioxide; ME, metabolizable energy; CP, crude protein; Lys, lysine; Met, methionine; Thr, threonine; Trp, tryptophan; Cys, cysteine; Ca, calcium; TP, total phosphorus.

**Table 4:** AIAADs of corn samples from different sources for medium-growing yellow-feathered broilers.

Item (%)	Corn Sources										Mean	Pooled SE	CV (%)	*p*-Value
	A1	A2	A3	A4	A5	A6	A7	A8	A9	A10				
EAAs														
AID Val	62.6 ^bc^	63.2 ^ab^	55.9 ^de^	63.7 ^ab^	57.1 ^d^	61.3 ^bcd^	60.8 ^bcd^	57.4 ^cd^	51.0 ^e^	68.4 ^a^	60.1	1.8	8.15	<0.001
AID Met	85.4 ^abc^	84.0 ^cd^	82.1 ^cde^	88.1 ^ab^	84.4 ^bcd^	85.7 ^abc^	80.8 ^de^	88.5 ^ab^	80.0 ^e^	88.8 ^a^	84.8	1.3	3.69	<0.001
AID Ile	76.0 ^ab^	70.5 ^bc^	67.6 ^c^	75.5 ^ab^	71.8 ^abc^	75.7 ^ab^	68.3 ^c^	77.8 ^a^	67.4 ^c^	78.5 ^a^	72.9	2.3	5.87	0.004
AID Leu	86.0 ^ab^	83.7 ^b^	83.2 ^bc^	87.6 ^ab^	84.4 ^ab^	85.7 ^ab^	78.6 ^c^	88.1 ^ab^	83.1 ^bc^	88.6 ^a^	84.9	1.7	3.52	0.006
AID Thr	58.8 ^abc^	57.7 ^bc^	52.1 ^c^	60.1 ^ab^	52.9 ^c^	53.7 ^bc^	52.9 ^c^	55.5 ^bc^	55.2 ^bc^	65.1 ^a^	56.4	2.2	7.21	0.004
AID Phe	75.4 ^abc^	74.5 ^bc^	69.4 ^de^	78.5 ^a^	71.5 ^cd^	75.7 ^ab^	65.9 ^e^	74.7 ^abc^	68.6 ^de^	77.7 ^ab^	73.2	1.3	5.66	<0.001
AID Lys	68.0 ^ab^	63.6 ^abc^	55.9 ^c^	61.9 ^abc^	60.0 ^bc^	64.6 ^ab^	60.5 ^bc^	68.2 ^ab^	61.4 ^bc^	69.5 ^a^	63.4	2.7	6.75	0.034
AID His	80.6 ^abc^	79.3 ^bc^	77.0 ^cd^	82.2 ^ab^	79.4 ^bc^	80.7 ^abc^	74.4 ^d^	83.2 ^ab^	73.6 ^d^	84.6 ^a^	79.5	1.6	4.55	<0.001
AID Arg	73.9 ^bcd^	75.8 ^abc^	72.3 ^cd^	77.5 ^ab^	70.0 ^de^	74.9 ^abc^	71.0 ^de^	72.8 ^cd^	67.8 ^e^	78.2 ^a^	73.4	1.4	4.49	<0.001
AID Trp	56.2 ^ab^	60.2 ^a^	55.5 ^abc^	55.9 ^abc^	55.6 ^abc^	49.1 ^cd^	42.8 ^d^	52.7 ^bc^	49.5 ^bcd^	50.2 ^bcd^	52.8	2.4	9.42	0.002
NEAAs														
AID Asp	70.5 ^ab^	66.9 ^bc^	59.3 ^d^	69.1 ^ab^	63.2 ^cd^	67.8 ^abc^	62.7 ^cd^	70.9 ^ab^	62.8 ^cd^	72.9 ^a^	66.6	2.0	6.63	<0.001
AID Ser	67.9 ^bcd^	68.7 ^abc^	60.6 ^e^	73.3 ^ab^	62.4 ^de^	69.5 ^ab^	62.4 ^de^	70.3 ^ab^	62.9 ^cde^	74.8 ^a^	67.3	2.1	7.34	<0.001
AID Glu	84.6 ^abc^	85.7 ^ab^	81.2 ^cde^	85.6 ^ab^	82.6 ^bcd^	84.7 ^abc^	78.0 ^e^	86.1 ^ab^	79.5 ^de^	86.6 ^a^	83.5	1.3	3.60	<0.001
AID Gly	67.2 ^bc^	67.7 ^abc^	61.8 ^d^	69.0 ^ab^	65.3 ^bcd^	68.3 ^ab^	63.2 ^cd^	69.6 ^ab^	64.8 ^bcd^	72.9 ^a^	67.0	1.8	4.91	0.004
AID Ala	82.5 ^abc^	83.5 ^abc^	79.3 ^cd^	84.0 ^ab^	81.0 ^bcd^	84.1 ^ab^	77.9 ^d^	85.9 ^a^	79.1 ^cd^	85.9 ^a^	82.3	1.6	3.47	0.004
AID Cys	66.7 ^bc^	70.9 ^ab^	67.6 ^bc^	70.7 ^ab^	65.9 ^bc^	69.2 ^bc^	63.5 ^c^	69.9 ^b^	65.1 ^c^	76.3 ^a^	68.6	2.0	5.32	0.006
AID Tyr	82.1 ^bcde^	82.4 ^bcde^	80.2 ^de^	85.5 ^abc^	81.1 ^cde^	84.1 ^abcd^	77.8 ^e^	86.6 ^ab^	80.9 ^cde^	87.7 ^a^	82.9	1.6	3.74	0.003
AID Pro	79.3 ^abc^	80.3 ^abc^	77.0 ^bcd^	82.2 ^ab^	77.8 ^bcd^	78.9 ^abc^	64.8 ^e^	75.8 ^cd^	72.4 ^d^	83.7 ^a^	77.2	1.8	7.01	<0.001

Values represent the means of 6 replicate cages (4 birds per replicate cage) (*n* = 6); different lowercase letters indicate significant differences (*p* < 0.05). Abbreviations: AIAADs, apparent ileal amino acid digestibilities; AID, apparent ileal digestibility; SE, standard error; CV, coefficient of variation; EAAs, essential amino acids; Val, valine; Met, methionine; Ile, isoleucine; Leu, leucine; Thr, threonine; Phe, phenylalanine; Lys, lysine; His, histidine; Arg, arginine; Trp, tryptophan; NEAAs, non-essential amino acids; Asp, aspartic acid; Ser, serine; Glu, glutamic acid; Gly, glycine; Ala, alanine; Cys, cysteine; Tyr, tyrosine; Pro, proline.

**Table 5:** SIAADs of corn samples from different sources for medium-growing yellow-feathered broilers.

Item (%)	Corn Sources										Mean	Pooled SE	CV (%)	*p*-Value
	A1	A2	A3	A4	A5	A6	A7	A8	A9	A10				
EAAs														
SID Val	96.4 ^a^	96.2 ^a^	94.5 ^a^	92.0 ^a^	92.2 ^a^	96.4 ^a^	96.1 ^a^	92.1 ^a^	86.4 ^b^	96.9 ^a^	93.9	1.8	3.53	0.004
SID Met	94.2 ^abc^	92.9 ^bcd^	92.2 ^bcd^	94.4 ^abc^	93.4 ^abcd^	95.4 ^ab^	90.1 ^d^	97.2 ^a^	91.1 ^cd^	95.5 ^ab^	93.6	1.3	2.30	0.021
SID Ile	93.8	87.7	88.1	90.6	90.8	94.1	87.6	96.1	86.1	93.6	90.9	2.3	3.75	0.054
SID Leu	94.9	92.3	93.6	95.0	94.1	94.8	88.8	97.2	92.7	96.2	94.0	1.7	2.49	0.089
SID Thr	90.4	88.9	87.9	86.4	86.3	86.2	86.9	88.2	88.9	92.8	88.3	2.2	2.36	0.567
SID Phe	94.3 ^a^	93.0 ^a^	91.5 ^ab^	94.0 ^a^	92.0 ^ab^	95.4 ^a^	87.3 ^c^	94.5 ^a^	88.9 ^bc^	94.1 ^a^	92.5	1.3	2.85	0.001
SID Lys	92.1	87.7	82.9	83.9	85.5	90.4	86.5	93.7	89.6	92.1	88.4	2.9	4.17	0.259
SID His	90.1 ^ab^	88.9 ^abc^	87.5 ^bcd^	90.1 ^ab^	89.3 ^ab^	90.4 ^ab^	84.5 ^cd^	92.5 ^a^	83.5 ^d^	92.6 ^a^	89.0	1.6	3.40	0.002
SID Arg	93.2 ^abcd^	95.4 ^ab^	93.8 ^abcd^	93.7 ^abc^	90.4 ^cd^	95.6 ^a^	91.4 ^bcd^	93.7 ^abcd^	89.8 ^d^	95.0 ^ab^	93.2	1.4	2.19	0.046
SID Trp	68.3 ^ab^	72.2 ^a^	68.0 ^ab^	65.6 ^abc^	67.7 ^abc^	62.1 ^bc^	54.8 ^d^	65.1 ^abc^	61.5 ^bcd^	60.0 ^cd^	64.5	2.4	7.75	0.002
NEAAs														
SID Asp	94.7 ^ab^	91.2 ^bc^	88.3 ^c^	90.6 ^bc^	90.2 ^bc^	94.2 ^abc^	89.8 ^bc^	97.7 ^a^	89.8 ^bc^	95.3 ^ab^	92.2	2.0	3.30	0.048
SID Ser	92.2	92.4	88.6	93.2	88.4	94.3	89.0	95.6	89.1	96.2	91.9	2.1	3.23	0.085
SID Glu	95.4 ^a^	96.4 ^a^	93.9 ^abc^	94.5 ^ab^	94.4 ^ab^	96.1 ^a^	90.3 ^c^	97.5 ^a^	91.4 ^bc^	95.9 ^a^	94.6	1.3	2.37	0.007
SID Gly	85.4	86.0	82.4	84.2	84.3	88.2	81.8	88.8	84.6	88.9	85.5	1.8	2.95	0.072
SID Ala	93.4	94.1	91.9	93.0	92.7	95.3	90.1	97.1	90.7	95.3	93.4	1.6	2.33	0.090
SID Cys	83.9	87.9	86.0	85.4	84.2	87.8	82.7	87.4	84.8	91.4	86.2	2.0	2.94	0.176
SID Tyr	92.2	92.1	91.6	93.3	91.9	94.4	89.1	96.6	91.7	96.2	92.9	1.6	2.44	0.095
SID Pro	89.1 ^ab^	89.8 ^ab^	88.2 ^ab^	90.0 ^ab^	88.1 ^ab^	89.2 ^ab^	75.6 ^d^	85.9 ^bc^	82.4 ^c^	92.1 ^a^	87.0	1.8	5.51	<0.001

Values represent the means of 6 replicate cages (4 birds per replicate cage) (*n* = 6); different lowercase letters indicate significant differences (*p* < 0.05). Abbreviations: SIAADs, standardized ileal amino acid digestibilities; SID, standardized ileal digestibility; SE, standard error; CV, coefficient of variation; EAAs, essential amino acids; Val, valine; Met, methionine; Ile, isoleucine; Leu, leucine; Thr, threonine; Phe, phenylalanine; Lys, lysine; His, histidine; Arg, arginine; Trp, tryptophan; NEAAs, non-essential amino acids; Asp, aspartic acid; Ser, serine; Glu, glutamic acid; Gly, glycine; Ala, alanine; Cys, cysteine; Tyr, tyrosine; Pro, proline.

**Table 6:** Correlations between chemical composition (air-dry basis) and SIAADs of corn samples for medium-growing yellow-feathered broilers.

Items (%)	CP (%)	EE (%)	CA (%)	CF (%)	ADF (%)	NDF (%)	PA (%)	NFE (%)	ST (%)
EAAs									
SID Val	−0.266	0.062	0.281	−0.103	−0.225	0.070	−0.038	0.340	−0.226
SID Met	0.154	−0.229	0.456	−0.425	−0.279	−0.152	−0.747 *	−0.278	−0.359
SID Ile	−0.048	−0.180	0.401	−0.385	−0.250	−0.140	−0.529	−0.067	−0.555
SID Leu	0.176	−0.191	0.395	−0.238	−0.168	−0.060	−0.832 *	−0.344	−0.345
SID Thr	0.464	0.127	0.461	0.084	0.381	0.471	−0.358	−0.209	0.170
SID Phe	−0.090	−0.372	0.301	−0.251	−0.539	−0.290	−0.799 *	0.012	−0.442
SID Lys	0.455	−0.407	0.366	−0.406	−0.034	−0.072	−0.337	−0.037	−0.194
SID His	0.045	−0.015	0.462	−0.241	−0.185	0.012	−0.748 *	−0.266	−0.375
SID Arg	0.127	−0.335	0.518	−0.501	−0.503	−0.192	−0.488	0.071	0.020
SID Trp	−0.185	−0.287	−0.489	0.424	−0.338	−0.423	−0.537	0.111	−0.432
NEAAs									
SID Asp	0.298	−0.287	0.485	−0.513	−0.143	−0.129	−0.493	−0.140	−0.323
SID Ser	0.382	−0.229	0.743 *	−0.644	−0.235	−0.037	−0.601	−0.260	0.052
SID Glu	0.140	−0.322	0.252	−0.243	−0.375	−0.246	−0.799 *	−0.155	−0.376
SID Gly	0.477	−0.367	0.592	−0.507	−0.236	−0.008	−0.782 *	−0.309	−0.109
SID Ala	0.305	−0.294	0.458	−0.478	−0.285	−0.160	−0.735 *	−0.284	−0.263
SID Cys	0.596	−0.079	0.772 *	−0.427	−0.040	0.320	−0.738 *	−0.479	0.280
SID Tyr	0.465	−0.174	0.666	−0.523	−0.100	0.063	−0.778 *	−0.497	−0.054
SID Pro	−0.057	−0.165	0.261	0.098	−0.351	0.002	−0.919 *	−0.110	−0.284

* Represents a significant difference (*p* < 0.05); The SIAAD values of 9 tested corn (*n* = 9) were used for correlation (r) analysis. Abbreviations: SIAADs, standardized ileal amino acid digestibilities; SID, standardized ileal digestibility; CP, crude protein; EE, crude fat; CA, crude ash; CF, crude fiber; ADF, acid detergent fiber; NDF, neutral detergent fiber; PA, phytic acid; NFE, nitrogen-free extract; ST, starch; EAAs, essential amino acids; Val, valine; Met, methionine; Ile, isoleucine; Leu, leucine; Thr, threonine; Phe, phenylalanine; Lys, lysine; His, histidine; Arg, arginine; Trp, tryptophan; NEAAs, non-essential amino acids; Asp, aspartic acid; Ser, serine; Glu, glutamic acid; Gly, glycine; Ala, alanine; Cys, cysteine; Tyr, tyrosine; Pro, proline.

**Table 7:** Correlations between EAAs (air-dry basis) and SIAADs of corn samples for medium-growing yellow-feathered broilers.

Items (%)	Val (%)	Met (%)	Ile (%)	Leu (%)	Thr (%)	Phe (%)	Lys (%)	His (%)	Arg (%)	Trp (%)
EAAs										
SID Val	0.171	0.024	0.007	0.210	0.337	0.073	0.373	0.204	0.330	0.096
SID Met	0.001	0.183	−0.050	0.204	0.184	−0.032	0.006	−0.005	0.168	0.293
SID Ile	−0.067	0.245	−0.129	0.084	0.127	−0.154	0.008	−0.004	0.249	0.154
SID Leu	0.088	0.147	0.031	0.286	0.234	0.066	0.107	0.095	0.238	0.366
SID Thr	0.204	0.387	−0.141	0.357	0.452	0.088	0.864 *	0.337	0.321	0.324
SID Phe	0.227	−0.124	0.199	0.322	0.288	0.180	0.148	0.169	0.359	0.296
SID Lys	−0.339	0.496	−0.540	−0.254	−0.140	−0.514	0.272	−0.177	−0.031	−0.179
SID His	0.132	0.092	0.046	0.372	0.361	0.119	0.169	0.105	0.283	0.403
SID Arg	0.277	0.081	0.119	0.348	0.396	0.189	0.326	0.241	0.288	0.321
SID Trp	0.065	−0.773 *	0.268	0.023	−0.105	0.151	−0.003	−0.172	0.034	−0.148
NEAAs										
SID Asp	−0.178	0.411	−0.321	−0.024	0.056	−0.305	0.158	−0.091	0.120	0.066
SID Ser	0.207	0.417	−0.003	0.385	0.433	0.109	0.327	0.245	0.292	0.476
SID Glu	−0.035	−0.092	−0.057	0.133	0.098	−0.048	0.076	−0.119	0.074	0.130
SID Gly	−0.167	0.403	−0.362	0.067	0.089	−0.250	0.177	−0.092	−0.045	0.176
SID Ala	−0.148	0.205	−0.228	0.073	0.072	−0.185	0.015	−0.162	−0.015	0.159
SID Cys	0.069	0.467	−0.230	0.372	0.389	0.029	0.405	0.134	0.005	0.470
SID Tyr	−0.020	0.442	−0.181	0.253	0.242	−0.059	0.124	0.031	0.066	0.406
SID Pro	0.397	−0.240	0.314	0.544	0.465	0.403	0.381	0.324	0.399	0.463

* Represents a significant difference (*p* < 0.05); the SIAAD values of 9 tested corn (*n* = 9) were used for correlation (r) analysis. Abbreviations: SIAADs, standardized ileal amino acid digestibilities; SID, standardized ileal digestibility; EAAs, essential amino acids; Val, valine; Met, methionine; Ile, isoleucine; Leu, leucine; Thr, threonine; Phe, phenylalanine; Lys, lysine; His, histidine; Arg, arginine; Trp, tryptophan; NEAAs, non-essential amino acids; Asp, aspartic acid; Ser, serine; Glu, glutamic acid; Gly, glycine; Ala, alanine; Cys, cysteine; Tyr, tyrosine; Pro, proline.

**Table 8:** Correlations between NEAAs (air-dry basis) and SIAADs of corn samples for medium-growing yellow-feathered broilers.

Items (%)	Asp (%)	Ser (%)	Glu (%)	Gly (%)	Ala (%)	Cys (%)	Tyr (%)	Pro (%)
EAAs								
SID Val	0.356	0.331	0.237	0.258	0.149	−0.090	−0.025	0.090
SID Met	0.074	0.120	0.080	0.153	0.084	0.068	0.424	0.053
SID Ile	0.032	0.052	−0.021	0.081	−0.047	0.119	0.406	−0.052
SID Leu	0.141	0.134	0.119	0.239	0.179	0.045	0.497	0.183
SID Thr	0.512	0.221	0.157	0.461	0.254	0.303	−0.229	0.371
SID Phe	0.274	0.293	0.284	0.294	0.254	−0.227	0.559	0.159
SID Lys	−0.086	−0.329	−0.386	−0.127	−0.390	0.462	−0.222	−0.297
SID His	0.243	0.302	0.237	0.301	0.255	−0.067	0.412	0.208
SID Arg	0.408	0.394	0.366	0.371	0.277	0.004	0.181	0.185
SID Trp	0.034	0.075	0.116	−0.052	0.103	−0.809 *	0.242	−0.072
NEAAs								
SID Asp	0.018	−0.063	−0.141	0.025	−0.172	0.310	0.103	−0.146
SID Ser	0.360	0.329	0.281	0.404	0.251	0.318	0.250	0.248
SID Glu	0.075	0.092	0.063	0.078	0.051	−0.200	0.253	−0.041
SID Gly	0.018	−0.099	−0.130	0.071	−0.085	0.334	0.026	−0.051
SID Ala	−0.015	−0.003	−0.049	0.029	−0.054	0.102	0.167	−0.092
SID Cys	0.276	0.164	0.134	0.348	0.226	0.390	−0.094	0.277
SID Tyr	0.099	0.075	0.042	0.213	0.102	0.350	0.237	0.141
SID Pro	0.457	0.422	0.434	0.485	0.499	−0.345	0.498	0.426

* Represents a significant difference (*p* < 0.05); The SIAAD values of 9 tested corn (*n* = 9) were used for correlation (r) analysis. Abbreviations: SIAADs, standardized ileal amino acid digestibilities; SID, standardized ileal digestibility; EAAs, essential amino acids; Val, valine; Met, methionine; Ile, isoleucine; Leu, leucine; Thr, threonine; Phe, phenylalanine; Lys, lysine; His, histidine; Arg, arginine; Trp, tryptophan; NEAAs, non-essential amino acids; Asp, aspartic acid; Ser, serine; Glu, glutamic acid; Gly, glycine; Ala, alanine; Cys, cysteine; Tyr, tyrosine; Pro, proline.

**Table 9:** SIAAD prediction models for corn samples in medium-growing yellow-feathered broilers (air-dry basis) *.

Prediction Equation	The Number of Predictor Variables	*R* ^2^	RMSE	*p*-Value
EAAs (%)				
SID Met = 120.05 − 2.85CF − 23.00PA	2	0.747	0.75	0.016
SID Phe = 122.41 − 1.91ADF − 27.23PA	2	0.782	1.49	0.010
SID His = 119.53 − 32.57PA	1	0.559	2.25	0.021
SID Trp = 52.10 + 3.56CP − 133.40Cys	2	0.883	0.02	<0.002
NEAAs (%)				
SID Glu = 119.03 − 26.08PA	1	0.639	1.52	0.010
SID Pro = 146.81 − 61.10PA	1	0.844	0.49	<0.001

* The SID values of 9 tested corn (*n* = 9) were used to construct the SIAAD prediction models. Abbreviations: SIAADs, standardized ileal amino acid digestibilities; SID, standardized ileal digestibility; CF, crude fiber (%); PA, phytic acid (%); ADF, acid detergent fiber (%); CP, crude protein (%); EAAs, essential amino acids; Met, methionine; Phe, phenylalanine; His, histidine; Trp, tryptophan; NEAAs, non-essential amino acids; Glu, glutamic acid; Cys, cysteine; Pro, proline; *R*^2^, coefficient of determination; RMSE, root mean square error.

**Table 10:** Preliminary assessment of the applicability of SIAAD prediction models for corn samples in medium-growing yellow-feathered broilers.

Item (%)	Determined Value of SIAAD in Corn A3 ^1^ (%)	Predicted Value of SIAAD in Corn A3 (%)	Difference Value ^2^ (%)	Accuracy Evaluation
SID Met	92.2 ± 1.2	92.6	0.4	Within
SID Phe	91.5 ± 0.9	92.4	0.9	Within
SID His	87.5 ± 3.2	88.7	1.2	Within
SID Trp	68.0 ± 1.6	72.6	4.6	Outside
SID Glu	93.9 ± 2.3	94.3	0.4	Within
SID Pro	88.2 ± 4.5	86.1	2.1	Within

^1^ The determined value is the mean ± standard deviation (SD) of 6 replicate values (*n* = 6). ^2^ The difference value equals the deviation between determined and predicted values. Absolute deviations between SIAAD predicted and measured values that fall within the SD of the measured values indicate relatively good agreement. Abbreviations: SIAADs, standardized ileal amino acid digestibilities; SID, standardized ileal digestibility; Met, methionine; Phe, phenylalanine; His, histidine; Trp, tryptophan; Glu, glutamic acid; Pro, proline. This comparison is descriptive and does not constitute formal model validation.

## Data Availability

The primary data supporting the outcomes of this investigation are available from the corresponding author when requested.
